# Inverse association of mortality and body mass index in patients with left ventricular systolic dysfunction of both ischemic and non‐ischemic etiologies

**DOI:** 10.1002/clc.23556

**Published:** 2021-03-06

**Authors:** Tiffany Brazile, Suresh Mulukutla, Floyd Thoma, N. A. Mark Estes, Sandeep Jain, Samir Saba

**Affiliations:** ^1^ Department of Medicine University of Pittsburgh Medical Center Pittsburg Pennsylvania USA; ^2^ Heart and Vascular Institute University of Pittsburgh Medical Center Pittsburg Pennsylvania USA

**Keywords:** left ventricular systolic dysfunction, morbidity, mortality, obesity

## Abstract

**Background:**

Obesity is a worldwide epidemic that has been associated with poor outcomes. Previous studies have demonstrated an inverse relationship between body mass index (BMI) and outcomes, the 'obesity paradox', in several diseases.

**Hypothesis:**

We sought to evaluate whether the obesity paradox is present in patients with left ventricular systolic dysfunction (LVSD) of all etiologies, using all‐cause mortality as the primary endpoint and hospitalization as the secondary endpoint.

**Methods:**

We conducted a retrospective cohort study of LVSD patients (*n* = 18 003) seen within the University of Pittsburgh Medical Center network between January 2011 and December 2017. Patients were divided into four BMI categories (underweight, normal weight, overweight, and obese) and stratified by left ventricular ejection fraction (LVEF): <20%, 20–35%, and 35–50%.

**Results:**

Over a median follow‐up of 2.28 years, higher BMI (mean 28.9 ± 6.8) was associated with better survival for the overall cohort and within LVEF strata (*p* < .0001). The most common cause of hospitalization was subendocardial infarction among underweight and normal weight patients and heart failure among overweight and obese patients. Cox proportional hazards model showed that BMI, age, and comorbid conditions of diabetes mellitus, chronic obstructive pulmonary disease, chronic kidney disease, and prior myocardial infarction are independent predictors of death.

**Conclusions:**

Our results support the existence of an obesity paradox impacting all‐cause mortality in patients with LVSD of ischemic and non‐ischemic etiologies even after adjusting for LVEF and comorbidities. Additional research is needed to understand the effect of weight loss on survival once a diagnosis of LVSD is established.

## 1 INTRODUCTION

Obesity has reached epidemic proportions in most developed countries affecting nearly every age and socioeconomic group. It is a risk factor for chronic noncommunicable diseases, including diabetes mellitus, hypertension, cardiovascular disease, and stroke.^1^ These health consequences lead to increased healthcare system costs, lost productivity, reduced quality of life, and premature death.^2^, ^3^, ^4^ Despite its association with poor outcomes, multiple studies have demonstrated an inverse relationship between body mass index (BMI) and patient outcomes, the so called “obesity paradox,” in several diseases.^5^, ^6^, ^7^ Among these diseases is heart failure (HF), which affects around 6.5 million adults in the United States^8^ with an anticipated 46% increase in prevalence by 2030.^9^

Although multiple investigations have demonstrated the counterintuitive decrease in mortality in patients with elevated BMI in the context of HF, results have been inconsistent or limited to specific patient subgroups.^10^, ^11^, ^12^, ^13^, ^14^, ^15^ Due to these conflicting results, we evaluated whether BMI is inversely proportional to mortality in a large contemporary cohort of patients with left ventricular systolic dysfunction (LVSD) of any etiology with a left ventricular ejection fraction (LVEF) ≤50%.

## 2 METHODS

This retrospective cohort study obtained data from the electronic health record of the University of Pittsburgh Medical Center (UPMC), a non‐for‐profit academic medical system in Pennsylvania with practice settings ranging from small rural hospitals to large urban quaternary centers, as previously described.^16^ Included in the cohort were adults aged ≥18 years with a documented LVEF ≤50% by echocardiogram from January 2011 to December 2017. The UPMC internal review board approved the study. The date of indexed outpatient UPMC facility visit served as the baseline date of entry to the cohort.

Patients were divided into three categories based on LVEF: <20%, 20–35%, and 36–50% and stratified according to BMI per World Health Organization definitions.^17^ LVEF categories were selected based on established thresholds that correlate with mortality as well as clinical indications for advanced therapies, such as intracardiac defibrillator implantation and heart transplantation.^18^, ^19^, ^20^ Patients were followed from the time of first documented LVEF≤50% within the UPMC system to the endpoint of death, hospitalization, or the end of the study period.

We present descriptive characteristics as means (± SD) for continuous variables and counts (%) for categorical variables. We performed comparisons between groups using *t* tests for continuous variables and *χ* ^2^ tests for categorical variables. BMI‐specific outcomes of LVSD patients including all‐cause mortality, any hospital admission, and cardiac hospital admission were compared within each LVEF stratum. Mortality was confirmed using the United States Social Security Death Index. Kaplan–Meier curves were created to evaluate for differences in mortality by BMI status. A Cox proportional hazards model was created to assess the independent predictive value of BMI on mortality. We considered all patient characteristics from Table 1 which are either known from the literature to impact mortality or that reached statistical significance with a *p*‐value <.05 between BMI groups. A two‐sided alpha level of .05 signified statistical significance. We used Stata software version 16.1 (StataCorp, College Station, TX, USA) to perform the analyses.

**TABLE 1:** Baseline characteristics according to body mass index categories among cardiomyopathy patients

Baseline characteristics of patients					
Characteristic	BMI < 18.5 (*N* = 467)	BMI 18.5–24.9 (*N* = 4459)	BMI 25–29.9 (*N* = 5535)	BMI > 30 (*N* = 7542)	*p*‐value
Age – mean ± SD (years)	70.3 ± 16.4	73.4 ± 14.8	71.8 ± 13.5	67.3 ± 13.8	<.0001
Female sex – no (%)	291 (62.3)	1835 (27.2)	1723 (25.6)	2889 (42.9)	<.0001
Race–no (%)					
White	399 (85.4)	3953 (88.7)	4975 (89.9)	6477 (85.9)	
Black	59 (12.6)	387 (8.7)	461 (8.3)	971 (12.9)	
Asian	1 (0.2)	28 (0.6)	11 (0.2)	17 (0.2)	
Other	5 (1.1)	48 (1.1)	44 (0.8)	57 (0.8)	
Medical history – no (%)					
Hypertension	204 (43.7)	2310 (51.8)	3177 (57.4)	4725 (62.7)	<.0001
Dyslipidemia	174 (37.3)	2143 (48.1)	3078 (55.6)	4159 (55.1)	<.0001
Diabetes mellitus	38 (8.1)	829 (18.6)	1566 (28.3)	2917 (38.7)	<.0001
Chronic obstructive pulmonary disease	138 (29.6)	694 (15.6)	765 (13.8)	1087 (14.4)	<.0001
Coronary artery disease	143 (30.6)	2030 (45.5)	2796 (50.5)	3393 (45.0)	<.0001
Atrial fibrillation	93 (19.9)	1128 (25.3)	1611 (29.1)	2085 (27.7)	<.0001
Stroke	44 (9.4)	594 (13.3)	757 (13.7)	857 (11.4)	<.0001
Myocardial infarction	37 (7.9)	388 (8.1)	484 (8.7)	554 (7.4)	.012
Chronic kidney disease	30 (6.4)	406 (9.1)	490 (8.9)	760 (10.1)	<.0001

## 3 RESULTS

During the study period we identified 18 003 unique patients with an LVEF ≤50% who had a total of 43 042 hospital admissions. Of this cohort, 71.4% of patients had at least one admission, and 8037 died (44.6% of patients) over a median follow‐up period of 3.35 years. Baseline characteristics of patients stratified by BMI are shown in Table 1. Most patients in this study were categorized as obese (41.9%), followed by overweight (30.7%), normal weight (24.8%), and underweight (2.6%). Mean age in years was 70.3 ± 14.3 in the total cohort. Patients in the obese group were significantly younger than the normal weight group (*p* < .0001). The mean LVEF of the total cohort was 31 ± 9%.

**FIGURE 1:**
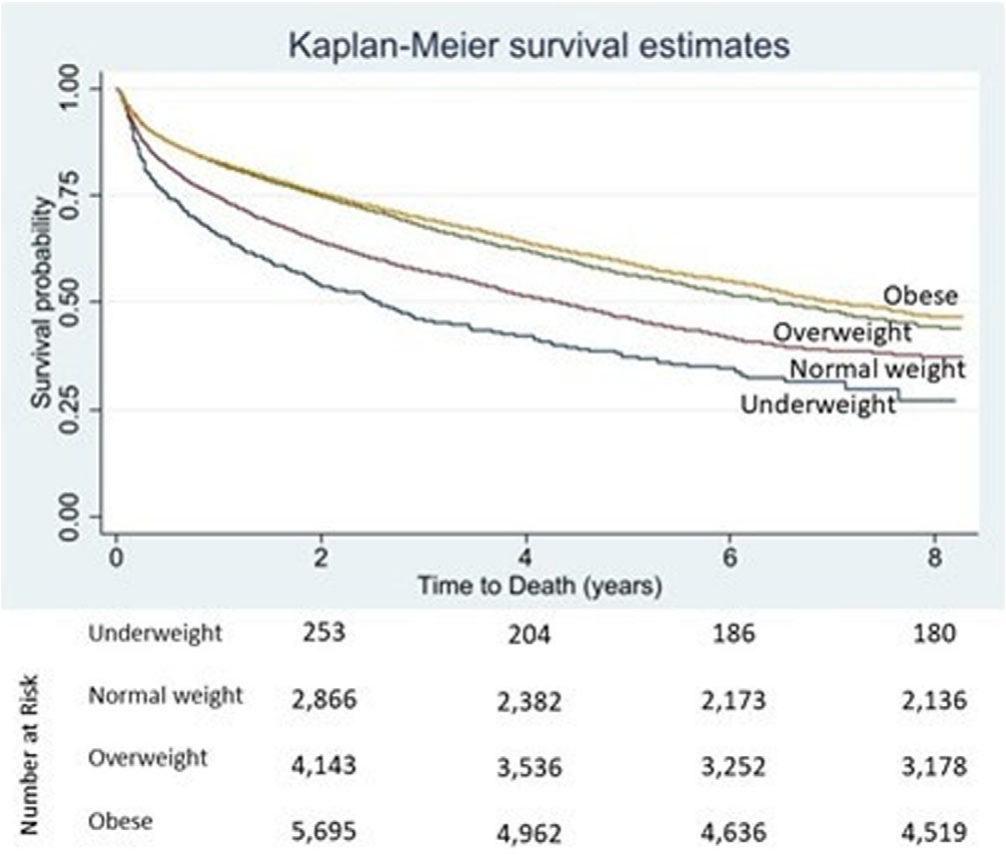
Kaplan–Meier survival curves for mortality stratified by BMI in cardiomyopathy patients (HR 0.82, 95% CI 0.80–0.85, p < 0.0001)

Mortality rates were higher in the underweight and normal weight groups relative to the overweight and obese groups. For every 1 kg/m^2^ increase in BMI, mortality decreased by 18% unadjusted and 12% adjusted (*p* < .0001 and *p* < .0001, respectively; Tables 2 and 3, Figure 1). An unadjusted Cox proportional hazards model for BMI demonstrated a statistically significant increase in mortality in the underweight group and decreased mortality in the overweight and obese groups relative to the normal weight group (Table 2). Kaplan–Meier survival curves by BMI group are shown in Figure 1. This trend persisted after adjusting for comorbidities shown in Table 3. The Cox proportional hazards model revealed that BMI and comorbid conditions of age, diabetes mellitus (DM) (Supplemental Table 1), chronic obstructive pulmonary disease (COPD), chronic kidney disease (CKD), and LVEF are independent predictors for death.

**TABLE 2:** Unadjusted Cox proportional hazards model for mortality stratified by BMI. Hazard ratio for all patients with BMI as a continuous variable as compared to BMI as a categorical variable with normal weight as the baseline

	HR	95% CI	*p*‐value
All patients	0.82	0.80–0.84	<.0001
Underweight	1.39	1.22–1.57	<.0001
Normal weight	1		
Overweight	0.75	0.71–0.80	<.0001
Obese	0.81	0.76–0.85	<.0001

**TABLE 3:** Multivariate Cox proportional hazards model with BMI as a continuous variable and adjusted for comorbidities including age, HTN, DM, COPD, CAD, CKD, LVEF, HLD, AFIB, MI, and stroke

	HR	95% CI	*p*‐value
BMI	0.9	0.87–0.92	<.0001
Age	1.04	1.04–1.05	<.0001
HTN	0.97	0.93–1.02	.309
DM	1.41	1.33–1.48	<.0001
COPD	1.6	1.50–1.68	<.0001
CAD	0.99	0.94–1.04	.652
CKD	1.58	1.48–1.69	<.0001
LVEF	0.98	0.97–0.98	<.0001
HLD	0.77	0.73–0.81	<.0001
AFIB	1.01	0.97–1.06	.594
MI	0.82	0.75–0.90	<.0001
Stroke	1.09	1.02–1.16	.01

Abbreviations: AFIB, atrial fibrillation; CAD, coronary artery disease; CKD, chronic kidney disease; COPD, chronic obstructive pulmonary disease; DM, diabetes mellitus; HLD, hyperlipidemia; HTN, hypertension; LVEF, eft ventricular ejection fraction; MI, myocardial infarction.

Median time to first hospitalization for the entire cohort was 262.8 days with a median time to first cardiac hospitalization of 390.6 days. Underweight and normal weight groups had shorter times to first hospitalization (98.6 and 146.1 days) than their overweight and obese counterparts (263.0 and 208.2 days). The comorbid chronic conditions of CKD, COPD, and DM had the strongest associations with shorter time to hospitalization (Table 4).

**TABLE 4:** Cox proportional hazards model for time to first hospitalization stratified by BMI and adjusted for comorbidities described in Table 3

	HR	95% CI	*p*‐value
Time to first hospitalization			
BMI			
Underweight	1.11	1.02–1.22	.04
Normal Weight	1.00		
Overweight	0.87	0.84–0.91	<.0001
Obese	0.95	0.91–0.98	.03
Age	1.02	1.01–1.02	<.0001
HTN	1.08	1.05–1.12	<.0001
DM	1.27	1.23–1.32	<.0001
COPD	1.34	1.28–1.40	<.0001
CAD	0.90	0.87–0.93	<.0001
CKD	1.51	1.43–1.60	<.0001
LVEF	0.99	0.98–0.99	<.0001
HLD	0.85	0.82–0.88	<.0001
AFIB	1.00	0.96–1.03	.90
MI	0.92	0.87–0.97	<0.0001
Stroke	1.15	1.10–1.21	<.0001

Abbreviations: AFIB, atrial fibrillation; CAD, coronary artery disease; CKD, chronic kidney disease; COPD, chronic obstructive pulmonary disease; DM, diabetes mellitus; HLD, hyperlipidemia; HTN, hypertension; LVEF, eft ventricular ejection fraction; MI, myocardial infarction.

## 4 DISCUSSION

Our results support the existence of an inverse relationship between BMI and risk of mortality or hospitalization in one of the largest cohorts of patients with LV systolic dysfunction of any etiology even when adjusting for the severity of LVSD. Our analysis shows that BMI is an independent predictor of mortality, although its influence is attenuated by comorbid conditions. Our findings show a U‐shaped relationship between BMI and mortality with a nadir of 32–33 kg/m^2^, as seen in other studies.^10^, ^12^ Stratification of the obese group into classes (class I: 30.0–34.9, class II: 35.0–39.9, class III: ≥40.0) as compared to the normal weight group suggest lower mortality in patients with class I obesity, further supporting the U‐shaped relationship previously described. Our results support the inverse relationship between BMI and mortality across all ages in contrast to other studies in which it was limited to older patients.^9^, ^10^

In contrast to the findings of Zamora et al,^11^ the protective effect of increased BMI in our cohort was not absent in patients with coexisting DM and was not limited to patients with non‐ischemic LVSD, although it was diminished as demonstrated in a subgroup analysis (Supplemental Table 1).

There have been multiple explanations proposed to account for the obesity paradox in LVSD patients. Obese patients are at increased risk to develop HF at a younger age, resulting in a better overall prognosis,^10^, ^21^ which may be due to lead‐time or survival bias from presentation at an earlier stage of disease and earlier initiation of therapy.^22^ However, the obesity paradox has been documented in both acute and chronic HF patients.^23^ Reverse causation may also play a role in the seeming improved survival in overweight and obese patients due to earlier diagnosis or even misdiagnosis of HF based on symptoms of dyspnea on exertion and orthopnea that may be due in part to body habitus.^24^ Our study focused on patients with systolic dysfunction on echocardiogram of any etiology, reducing the likelihood of misdiagnosis. Some studies suggest that the effect of BMI on mortality is attenuated by longer‐term follow up as well as improvements in therapeutic interventions,^25^, ^26^ however, patients with LVSD are at increased risk of developing overt HF^27^ and approximately half of patients who develop HF die within 5 years of diagnosis.^28^

Obesity may be protective once a patient has developed HF. Increased adiposity may serve as an energy reserve and delay the cachectic effects of HF via preservation of muscle mass and bone density.^29^, ^30^, ^31^ Since BMI is a surrogate measure of adiposity and does not reflect body composition, it does not identify patients with sarcopenic obesity who have decreased cardiac function and exercise capacity related to muscle loss.^32^, ^33^ Peak oxygen consumption, an independent predictor of HF outcomes, does not vary between obese and leaner patients with HFrEF when corrected for skeletal muscle mass suggesting that lean mass improves outcomes due to superior cardiorespiratory fitness levels.^34^ Measures of peak oxygen consumption were not included in this study, which would have provided more information about the impact of cardiorespiratory fitness on prognosis in relation to BMI. While there are concerns about the accuracy of BMI as a measure of adiposity, several studies demonstrate that alternative measures, including waist‐to‐hip ratio, waist circumference, and body fat percentage, do not better predict mortality.^35^, ^36^, ^37^, ^38^ As there are no clinical trials exploring weight loss in HF patients its effect on survivability is remains unknown. Nonetheless, unintentional weight loss in HF patients is a poor prognostic sign, even before patients appear cachectic.^39^

While several analyses refute the existence of an obesity paradox ascribing the results to collider bias,^40^, ^41^, ^42^, ^43^ there are plausible physiological explanations for the existence of this paradox based on energy balance, timing of diagnosis, frequency, volume, and intensity of care delivered, and differences in comorbidities among BMI classes.

The results of time to first hospitalization mirror the mortality findings suggesting that those with a higher BMI have a better functional status than those with normal or lower BMI. This could be due to factors such as younger age, greater skeletal muscle mass, increased outpatient monitoring due to elevated BMI, or more intensive management. As this study did not capture New York Heart Association functional classes, it is difficult to ascertain the degree to which functional status played a role in hospitalization. Consistent with other studies, the underweight group had the poorest prognosis with respect to mortality and time to hospitalization.^44^, ^45^ This is likely multifactorial due to not only the cachectic pathophysiology of HF,^46^ but also the increased prevalence of coexisting lung disease predisposing this group to COPD exacerbation and pneumonia.^47^

The clinical characteristics among the BMI groups in our cohort differ significantly, which may account for some of the differences in survival. However, such heterogeneity may increase the predictive value of the total cohort allowing greater applicability to the population at large as well as comparison with other LVSD cohorts despite this being a single center study. Weight was measured at a single point in time; thus, this study does not capture how changes in weight may modulate risk in LVSD patients. We do not have information about treatment intensity, nor care outside of our system, which may affect hospitalizations and ultimately, mortality. Comorbid diagnoses were collected from clinical histories and do not account for severity of illness, treatment regimens, or success of management.

This study does not explore the role of social determinants of health or the nutritional status of patients, which may influence morbidity and mortality. Other studies suggest an increased prevalence of obesity in lower socioeconomic groups, thus, it would be important to understand if the obesity paradox persists when accounting for social factors, by impacting time to diagnosis, access to care, and adherence to treatment.

This large cohort study demonstrates the existence of an inverse relationship between BMI and mortality in patients with LV systolic dysfunction of any etiology, even after accounting for comorbidities. This finding may have important implications to the caloric management of patients with LVSD. A study that monitors fluctuations in weight, body composition, and nutritional status over the course of disease may provide insight into the mechanisms by which obesity can be protective. Further research is needed to explore the influence of social determinants of health on BMI, healthcare utilization, and mortality in LVSD patients.

## CONFLICT OF INTEREST

Tiffany L. Brazile: No relationships to disclose. Suresh Mulukutla: No relationships to disclose. Floyd Thoma: No relationships to disclose. N. A. Mark Estes III, MD: Dr. Estes has consulted for Boston Scientific and Medtronic. Sandeep K. Jain: Dr. Jain reports research support from Abbott, Boston Scientific, and Medtronic. Samir Saba: Dr. Saba reports research support from Abbott, Boston Scientific, and Medtronic.

## Supporting information


**Supplemental Table 1** Cox Proportional Hazards Model for Diabetics and Non‐Diabetics stratified by BMI and left ventricular ejection fraction (LVEF). Cox Proportional Hazards Model for obese patients stratified by obesity class and adjusted for comorbidities as described in Table 3.

## Data Availability

Data available on request from the authors.
