# Supplementary material for: Inverse association of mortality and body mass index in patients with left ventricular systolic dysfunction of both ischemic and non‐ischemic etiologies
Source: Clin Cardiol. 2021 Mar 6;44(4):495–500. doi: 10.1002/clc.23556 (PMC8027570; doi:10.1002/clc.23556)
Supplement: Supplementary file 1 — Supplemental Table 1 Cox Proportional Hazards Model for Diabetics and Non‐Diabetics stratified by BMI and left ventricular ejection fraction (LVEF). Cox Proportional Hazards Model for obese patients stratified by obesity class and adjusted for comorbidities as described in Table 3. [file CLC-44-495-s001.docx]

Supplemental Table 1: Cox Proportional Hazards Model for Diabetics and Non-Diabetics stratified by BMI and left ventricular ejection fraction (LVEF). Cox Proportional Hazards Model for obese patients stratified by obesity class and adjusted for comorbidities as described in Table 3.
